# Interactions between Environmental Factors and Glutathione S-Transferase (GST) Genes with Respect to Detectable Blood Aluminum Concentrations in Jamaican Children

**DOI:** 10.3390/genes13101907

**Published:** 2022-10-20

**Authors:** Mohammad H. Rahbar, Maureen Samms-Vaughan, Yuansong Zhao, Sepideh Saroukhani, Jan Bressler, Manouchehr Hessabi, Megan L. Grove, Sydonnie Shakespeare-Pellington, Katherine A. Loveland

**Affiliations:** 1 Department of Epidemiology, Human Genetics, and Environmental Sciences (EHGES), School of Public Health, The University of Texas Health Science Center at Houston, Houston, TX 77030, USA; 2 Biostatistics/Epidemiology/Research Design (BERD) Component, Center for Clinical and Translational Sciences (CCTS), The University of Texas Health Science Center at Houston, Houston, TX 77030, USA; 3 Division of Clinical and Translational Sciences, Department of Internal Medicine, McGovern Medical School, The University of Texas Health Science Center at Houston, Houston, TX 77030, USA; 4 Department of Child & Adolescent Health, The University of the West Indies (UWI), Mona Campus, Kingston 7, Jamaica; 5 Department of Biostatistics & Data Science, School of Public Health, The University of Texas Health Science Center at Houston, Houston, TX 77030, USA; 6 Human Genetics Center, School of Public Health, The University of Texas Health Science Center at Houston, Houston, TX 77030, USA; 7 Louis A Faillace, MD, Department of Psychiatry and Behavioral Sciences, McGovern Medical School, The University of Texas Health Science Center at Houston, Houston, TX 77054, USA

**Keywords:** interaction, environmental factors, glutathione S-transferase (GST) genes, blood Aluminum concentrations, detoxification, Jamaican children

## Abstract

Aluminum (Al) is a metallic toxicant at high concentrations following natural or unnatural exposures. Dietary intake is considered as the main source of aluminum exposure in children. We used data from 366 typically developing (TD) children (ages 2–8 years) who participated as controls in an age- and sex-matched case–control study in Jamaica. We investigated additive and interactive associations among environmental factors and children’s genotypes for glutathione S-transferase (GST) genes (*GSTT1*, *GSTM1*, *GSTP1*), in relation to having a detectable blood aluminum concentration (BAlC) of >5.0 μg/L, using multivariable logistic regression models. Findings from interactive models revealed that the odds of having a detectable BAlC was significantly higher among children who ate string beans (*p* ≤ 0.01), whereas about 40% lower odds of having a detectable BAlC was observed in children with higher parental education level, (*p* = 0.02). A significant interaction between consumption of saltwater fish and *GSTP1* in relation to having a detectable BAlC using either co-dominant or dominant genetic models (overall interaction *p* = 0.02 for both models) indicated that consumption of saltwater fish was associated with higher odds of having a detectable BAlC only among children with the *GSTP1* Ile105Val Ile/Ile genotype using either co-dominant or dominant models [OR (95% CI) = 2.73 (1.07, 6.96), *p* = 0.04; and OR (95% CI) = 2.74 (1.08, 6.99), *p* = 0.03]. Since this is the first study from Jamaica that reports such findings, replication in other populations is warranted.

## 1. Introduction

Aluminum (Al) is one of the most plentiful elements after oxygen and silicon in the surface of the Earth and is about 8% by mass [1,2,3]. Even though Al is not required for any biological process in humans and animals, it can be a metallic toxicant at high concentrations after natural or unnatural exposure [4,5]. Exposure to Al has been linked to several adverse health effects, such as asthma [6,7], bone disease [8,9,10], immunotoxicity [11,12], congenital malformations [13], and reproductive toxicity in laboratory animals [14,15]. For example, children with early life exposure to a high level of Al had lower lumbar spine bone mass and lower hip bone mass [10]. Moreover, Al is associated with neurological diseases, including Alzheimer’s dementia and multiple sclerosis [16,17], and autism spectrum disorder (ASD) [18]. Specifically, several studies have shown that compared to people without recognizable neurological diseases, people with such disease had a significantly higher content of Al in brain tissue [16].

Major sources of human exposure to Al include food, while minor exposures to Al may occur through drinking water, occupational inhalation of ambient air, skin absorption of cosmetic products, parenteral nutrition solutions, and pharmaceutical products [19,20,21,22,23,24]. In addition to food items that naturally contain Al by uptake from the geologic surroundings during growth, the use of food additives such as firming, coloring, or anticaking agent that contain Al has obviously increased Al exposure in humans [24,25,26]. Many foods or food products have been reported to have high Al contents. For example, raw tea, which is made from young leaves, contains a reasonably high content of Al, and fermented tea has an even higher Al content [27]. A recent study in an Italian population suggests that legumes, sweets (mainly in chocolate-based products), cereals and cereal products, and leafy vegetables have high Al contents [26]. Furthermore, fish and shellfish are often considered as sources of dietary exposure to Al. The Second French Total Diet Study (TDS2) that was conducted by the French Food Safety Agency (AFSSA) reported that fish and fish products were the food groups that had the highest mean contents of Al [28]. As a result of industrialization, food packaging materials made from Al, such as Al cans and Al foil can also increase Al exposure in humans [26,29,30]. For example, even from the same dairy plant, processed cheese wrapped in Al foil had higher Al content than cheese packed in non-Al material (0.034 to 5.718 compared to 0.077 to 2.939 mg/kg) [30]. In its report in 2011, the Joint Food and Agriculture Organization of the United Nations/World Health Organization Expert Committee on Food Additives (JECFA) had proposed a provisional tolerable weekly intake (PTWI) of 2 mg/kg body weight (bw) that applies to all Al compounds in food, including food additives [31]. When expressed as body weight, the food intake of children is generally higher than that of adults, and therefore children are more susceptible to potential exposure to Al through diet. In fact, several studies suggest that dietary Al exposures are likely to be exceeded to a large extent in children in different countries and regions [32,33,34,35]. In Shenzhen, China, although the average dietary exposure to Al of the whole population is lower than the PTWI (1.263 mg/kg bw per week), children aged from 3–13 years have an Al intake of 3.248 mg/kg bw per week, which is 60% more than the PTWI suggested by JECFA [34]. Similarly, a study from Japan reported that the mean dietary intake of Al through food among children (2.85 mg/kg bw per week) was 40% higher than the PTWI in comparison with adults (1.37 mg/kg bw per week) [32]. Considering that Jamaica is one of the world’s major exporters of bauxite, and has a high per capita fish consumption, higher exposure to Al through dietary intake can pose a health risk in Jamaican children [36,37,38,39].

The glutathione S-transferase (GST) superfamily includes six genes of which GST pi 1 (*GSTP1*), GST mu 1 (*GSTM1*), and GST theta 1 (*GSTT1*) are phase II enzymes and have been known for their critical role in detoxification and excretory mechanisms [40]. These enzymes catalyze and promote excretion after conjugation of glutathione with numerous xenobiotics (e.g., heavy metals including Al) [41,42,43]. In several animal studies, significantly decreased GST activities and reduced GSH levels were found following Al exposure [44,45]. A similar result was obtained in a study by Halatek et al. of industrial workers in which the authors noted that people with higher concentrations of Al in urine (>40 μg/L) had a lower GST activity [46]. Moreover, several findings suggested that the differential susceptibility to heavy metals can be explained by the polymorphisms of GST genes, for instance, null alleles of *GSTT1* and *GSTM1* were associated with a deficiency of enzymatic activity which was related to decreased detoxification and increased oxidative stress [43,47]. According to a recent study in Egypt, children with null *GSTM1* and *GSTT1* genotypes had a significantly lower level of GST activity compared to other combinations of genotypes, suggesting a poor aluminum detoxification ability [48]. All of these reports indicate that genetic variation can provide an explanation for differences in Al concentrations in a population.

In our previous paper, we used data from 116 age- and sex-matched pairs (ASD vs. typically developing controls (TD)) (232 children) of Jamaican children 2–8 years old. We observed that TD children with *GSTP1* Ile/Ile or Val/Val genotype had a significantly higher geometric mean blood Al concentration (BAlC) than those with Ile/Val genotypes (23.75 μg/L vs. 14.57 μg/L, *p* < 0.03). Furthermore, none of the additive effects of food consumption were statistically significantly associated with log-transformed BAlC (all *p* > 0.06) [49]. In the present study, we evaluated the additive and interactive association between environmental factors and genotypes of three GST genes, as well as the possible pairwise gene-gene interactions of these genes in relation to a detectable BAlC (>5.0 μg/L) in Jamaican TD children.

## 2. Materials and Methods

### 2.1. Study Population

This study was conducted using data from 366 TD control children, between 2–8 years old, who were enrolled in the Epidemiological Research on Autism in Jamaica (ERAJ) studies between December 2009 and September 2017. Detailed information regarding the enrollment and assessment of TD controls has been reported earlier [50,51,52]. Relevant to the research objectives here, the age- and sex-matched TD controls (within six months of the matched ASD case) were identified from schools, churches, and well-child clinics at the University of the West Indies (UWI) and the Social Communication Questionnaire (SCQ) [53] was used to rule out developmental disorders (SCQ score of 0–6) in the TD control children. [49] We collected information about parents/guardians’ sociodemographic characteristics as well as children’s weekly food intake through questionnaires [49], and about 5 mL of whole blood was drawn from each child to assess exposure to the heavy metals including Al and to determine genotypes for the three GST genes. This study was approved by the Institutional Review Boards of The University of Texas Health Science Center in Houston (UTHealth), Michigan Department of Health and Human Services (MDHHS), and the University of the West Indies, Mona campus, in Kingston, Jamaica (HSC-SPH-09-0059).

### 2.2. Assessment of Al Exposure

In this study, we assessed BAlCs to measure Al exposure in children. BAlCs were assessed at the Trace Metals Lab at the MDHHS in Lansing, MI, USA. We have previously reported details on sample processing and storage [49,51,54]. MDHHS followed a fully authenticated protocol for analyzing Al in blood samples with a limit of detection (LoD) of 5.0 μg/L, and 37.1% (136 out of 366) of children in this study had an undetectable BAlC because it was below the LoD.

### 2.3. Statistical and Genetic Analysis

We conducted descriptive analyses to assess socioeconomic status (SES) characteristics, and frequencies of the *GSTP1*, *GSTT1*, and *GSTM1* genotypes for the TD children. Since more than one-third (37%) of BAlCs were below the LoD, we used 5.0 μg/L as the cutoff point and converted BAlCs to a binary variable. The choice of cutoff point reflects the LoD in the ERAJ studies.

Assessment of the children’s genotypes for the *GSTP1* Ile105Val (rs1695) polymorphism and insertion deletion polymorphisms in *GSTT1* and *GSTM1* was carried out as previously described [50,55]. Choice of the genetic models that were used to test their additive and interactive associations with environmental factors was based on differences in the types of polymorphisms. Because there are 3 possible genotypes for *GSTP1* rs1695 and 2 possible genotypes for *GSTT1* and *GSTM1* since the homozygote (I/I) and heterozygote (I/D) cannot be distinguished, only the recessive model was selected for *GSTT1* and *GSTM1* (D/D vs. I/I and I/D) whereas three different genetic models were specified for *GSTP1* rs1695 (dominant, co-dominant, and recessive). Similarly, only the *GSTP1* Ile105Val polymorphism was tested for accordance with Hardy–Weinberg equilibrium expectations using the Chi-square test.

Using logistic regression models, we assessed additive association of each independent variable including the three GST genes, sociodemographic characteristics, and consumption of different kinds of vegetables, starches, and seafoods in relation to binary BAlCs (<LoD vs. ≥LoD). Then, we evaluated the potential gene-gene interactions among the three GST genes and possible gene-environment interactions between each of the three GST genes and consumption of various types of food in relation to BAlCs. Subsequently, we developed logistic regression models that contained both additive and interactive effects of GST genes and environmental factors to evaluate the adjusted odds of having a detectable BAlC. To minimize the potential effects of multicollinearity, we only kept one of the correlated variables when the model became unstable by adding both correlated variables. Following the procedure described we used the CONTRAST statement in SAS [56] to access odds ratios and 95% confidence intervals for evaluating the interactive effects in the presence of two-way interactions. All statistical tests were evaluated at 5% level of significance using SAS 9.4 software [57].

## 3. Results

Demographic information and other characteristics are displayed in Table 1. 81.7% of the 366 TD children were male and 97.3% were Afro-Caribbean. About 25% of them were 72 months or older and 62.6% of the children were born in the Kingston parish. 11.4% of TD children were born to mothers who were at least 35 years old, and 45.5% of the children had at least one parent who attained an education level beyond high school. Moreover, 40.7% of the families owned a car, which represents high SES in Jamaica. The frequencies of null (DD) genotype for *GSTM1* and *GSTT1* were 26.1% and 24.6%, respectively. In addition, the frequencies of the *GSTP1* genotypes were in agreement with Hardy–Weinberg equilibrium expectations (*p* = 0.67).

In univariable logistic regression analysis (Table 2), we found a significant inverse association between having at least one parent with education level beyond high school and a detectable BAlC in children [OR (95% CI) = 0.52 (0.34, 0.80), *p* < 0.01]. We also found significant associations between consumption of certain types of food and BAlCs.

Specifically, the odds of having a detectable BAlC in children who ate green banana was significantly lower than in children who never ate such food [OR (95% CI) = 0.59 (0.36, 0.95), *p* = 0.03]. Our findings were similar for consumption of tuna [OR (95% CI) = 0.64 (0.41, 0.99), *p* = 0.04], cabbage [OR (95% CI) = 0.50 (0.32, 0.80), *p* < 0.01], and root vegetables (yam, sweet potato, dasheen, coco) [OR (95% CI) = 0.60 (0.37, 0.97), *p* = 0.03]] in relation to a detectable BAlC. Furthermore, the odds of having BAlCs above LoD were higher in children who consumed fresh water fish [OR (95% CI) = 1.80 (1.11, 2.91), *p* = 0.02], cakes/buns [OR (95% CI) = 1.87 (1.05, 3.32), *p* = 0.03], and lettuce [OR (95% CI) = 1.84 (1.19, 2.84), *p* < 0.01] than in those who did not eat these foods. Furthermore, children who consumed broad beans [OR (95% CI) = 1.84 (1.19, 2.83), *p* < 0.01], string beans [OR (95% CI) = 3.27 (2.05, 5.22), *p* < 0.01], as well as other beans and legumes (red and gungo peas) [OR (95% CI) = 1.96 (1.18, 3.27), *p* = 0.01], had higher odds of having a detectable BAlC compared to those who never ate such food. We did not find any significant additive associations between BAlCs and genotypes for the three GST genes (all *p* > 0.08).

Unadjusted multivariable models were used to assess the two-way gene-gene interaction of GST genes in relation to BAlCs (Table 3). Using a dominant genetic model for *GSTP1*, there was a significant interaction between *GSTP1* and *GSTM1* with respect to BAlCs (overall interaction *p* = 0.04) indicating that among children with *GSTM1* DD genotype, children with *GSTP1* Ile/Val or Val/Val genotype were 68% less likely to have a detectable BAlC than those with the *GSTP1* Ile/Ile genotype [OR (95% CI) = 0.32 (0.11, 0.98), *p* < 0.05].

Additionally, using a co-dominant model for *GSTP1*, although the interaction between *GSTP1* and *GSTM1* was marginally significant (overall interaction *p* = 0.06), we found that among children with *GSTM1* DD genotype, the odds of having a detectable BAlC in children with the *GSTP1* Val/Val genotype was 0.20 times (or 1/5 times) that of those with the Ile/Ile genotype [OR (95% CI) = 0.20 (0.05, 0.82), *p* = 0.03]. When we used the recessive genetic model for *GSTP1* (overall interaction *p* = 0.10), we found that (though marginally significant) among children with *GSTP1* Val/Val genotype, the odds of having a detectable BAlC in children with the *GSTM1* DD genotype was 0.33 times that of those with the I/I or I/D genotype [OR (95% CI) = 0.33 (0.10, 1.07), *p* = 0.06]. Moreover, although the interaction between *GSTM1* and *GSTT1* in relation to BAlCs was not statistically significant (overall interaction *p* = 0.11), we found among children with DD genotype for *GSTM1*, the odds of having a detectable BAlC was 67% lower in children with DD genotype for *GSTT1* than in those with I/I or I/D genotype for *GSTT1* [OR (95% CI) = 0.33 (0.13, 0.87), *p* = 0.03].

In the assessment of the interactive associations of children’s environmental exposures and genotypes for GST genes with respect to detectable BAlCs (Table 4), we identified a significant interaction between consumption of green banana and *GSTT1* genotypes in relation to BAlCs (interaction *p* = 0.04). Specifically, using a recessive genetic model, among children with *GSTT1* I/I or I/D genotype, the odds of having a BAlC above LoD in children who ate green banana was 0.45 times that of those who never ate such food [OR (95% CI) = 0.45 (0.25, 0.82), *p* = 0.01], whereas, no statistically significant associations were found between consumption of green banana and BAlCs among children with *GSTT1* DD genotypes [OR (95% CI) = 1.47 (0.55, 3.90), *p* = 0.44]. In addition, we found a similar interactive association between child’s genotypes for *GSTT1* and consumption of porridge, as well as consumption of macaroni in relation to BAlCs (both overall interaction *p* = 0.03). For example, consumption of porridge or macaroni was associated with about 80% lower odds of having a detectable BAlC among children with *GSTT1* I/I or I/D genotypes [OR (95% CI) = 0.27 (0.10, 0.73), *p* = 0.01, and OR (95% CI) = 0.22 (0.05, 0.97), *p* < 0.05, respectively], whereas, consumption of porridge or macaroni was not statistically associated with BAlCs among children with *GSTT1* DD genotypes [OR (95% CI) = 1.51 (0.46, 4.91), *p* = 0.49, and OR (95% CI) = 1.97 (0.52, 7.52), *p* = 0.32, respectively].

Furthermore, there was a significant interaction between consumption of broad beans and *GSTM1* genotypes under a recessive genetic model, in relation to BAlCs (interaction *p* < 0.05). Specifically, among children with *GSTM1* null (DD) genotype, the odds of having a detectable BAlC in those who ate broad beans was 3.96 times that of those who never ate such food [OR (95% CI) = 3.96 (1.57, 9.97), *p* < 0.01], whereas, there was no significant association between consumption of broad beans and BAlCs among children with *GSTM1* I/I or I/D genotypes [OR (95% CI) = 1.37 (0.82, 2.27), *p* = 0.23]. We also identified a significant interaction between consumption of saltwater fish and child’s *GSTP1* genotype in relation to a detectable BAlC using either a co-dominant or dominant genetic model (overall interaction *p* = 0.03, and *p* = 0.02, respectively). Specifically, among children with *GSTP1* Ile/Ile genotypes, the odds of having detectable BAlCs in children who reported eating saltwater fish was 3.36 times that of those who never ate such seafood in both genetic models [OR (95% CI) = 3.36 (1.37, 8.24), *p* = <0.01 for both models]. Although the overall interaction was marginally significant when using the recessive genetic model (overall interaction *p* = 0.05), we have found that among children with at least one Ile allele, those who ate saltwater fish had higher odds of having detectable BAlCs than those who never ate such food [OR (95% CI) = 1.76 (1.05, 2.94), *p* = 0.03]. In a dominant model for *GSTP1*, we have also found a significant interaction between children’s genotypes for *GSTP1* and consumption of white bread in relation to BAlCs (overall *p* = 0.02). Specifically, among children with *GSTP1* Ile/Ile genotype, children who ate white bread were 2.49 times more likely to have detectable BAlCs compared to children who never ate white bread [OR (95% CI) = 2.49 (1.05, 5.90), *p* = 0.04]. This association was not statistically significant in children with *GSTP1* Ile/Val or Val/Val genotypes [OR (95% CI) = 0.75 (0.43, 1.29), *p* = 0.30]. Furthermore, among children with *GSTP1* Ile/Val or Val/Val genotypes, children who ate whole wheat bread were 2.05 times more likely to have detectable BAlCs compared to children who never ate such food [OR (95% CI) = 2.05 (1.22, 3.45), *p* < 0.01, overall interaction *p* = 0.02], whereas no statistically significant associations were found between consumption of whole wheat bread and BAlCs among children with *GSTP1* Ile/Ile genotype [OR (95% CI) = 0.59 (0.23, 1.52), *p* = 0.27]. Additional results for the unadjusted associations between genotypes for GST genes and BAlCs by exposure to environmental and dietary factors in TD children are shown in the Supplemental Materials (Table S1).

In the interactive multivariable models that assessed the adjusted associations of children’s GST genotypes, exposure to environmental factors, and their interactions in relation to a detectable BAlC (Table 5), we identified education level of the parents and consumption of string beans as environmental factors that are additively associated with BAlCs in Jamaican TD children (all *p* ≤ 0.02 in all models). For example, using the co-dominant model for *GSTP1* genotype, the odds of having detectable BAlCs in children who consumed string beans was still significantly higher than in those who never ate string beans (OR (95% CI) = 3.07 (1.07, 5.09), *p* < 0.01), and having at least one parent with education level beyond high school versus up to high school was associated with significantly lower odds of having a BAlC above LoD [OR (95% CI) = 0.57 (0.36, 0.91), *p* = 0.02]. By using three genetic models for *GSTP1* genotype, we have investigated the gene-environment interaction between *GSTP1* and consumption of saltwater fish in relation to BAlCs. After holding the aforementioned environmental factors constant, we found similar significant interactions between consumption of saltwater fish and *GSTP1* under both co-dominant and dominant genetic models (overall interaction *p* = 0.02 for both models). Specifically, we found that among children with *GSTP1* Ile/Ile genotype, the odds of having detectable BAlCs in children who ate saltwater fish was about 2.7 times that of those who never ate saltwater fish based on both co-dominant or dominant genetic models [OR (95% CI) = 2.73 (1.07, 6.96), *p* = 0.04; and OR (95% CI) = 2.74 (1.08, 6.99), *p* = 0.03, respectively]. Though the overall interaction is significant (*p* = 0.04), no statistically significant associations between saltwater fish consumption and BAlCs by children’s genotypes in *GSTP1* was found using the recessive genetic model for *GSTP1*. In addition, details about adjusted associations between children’s genotypes in *GSTP1* and BAlCs by saltwater fish consumption are shown in Table S2.

## 4. Discussion

Findings from our study suggest that the odds of having a detectable BAlC was about 3 times higher among children who ate string beans, compared to those who did not eat such beans, and 50% times lower in children with at least one parent with education level beyond high school. The association between consumption of saltwater fish and having a detectable BAlC varied by children’s genotype for *GSTP1* using either dominant or co-dominant genetic models (overall interaction *p* = 0.02 for both models), and eating saltwater fish was significantly associated with 3 times higher odds of having a detectable BAlC only among children with *GSTP1* Ile/Ile genotype.

Jamaica is known for its abundant and high-quality bauxite for decades. Over 20% of the surface area was covered by bauxite deposits in Jamaica, and a comparatively high level of Al was found in soils [37,59]. Since content of Al in foods varies by species and the soil pH [60], a possible explanation for our finding that consumption of string beans is associated with higher odds of having a detectable BAlC is that legumes including string beans accumulate more Al than others. Filippini et al. [26] conducted a study about dietary intake of Al by obtaining 908 food samples from Italy and measuring the Al content. Legumes were the category of food that had the highest levels of Al (7370.23 μg/kg). In addition, our finding is similar to several studies in China that reported soybeans, a member of the legume family, and bean products had a higher level of dietary Al content [34,61,62].

Our finding indicating 50% lower odds of having a detectable BAlC in children who had parents with higher education levels (at least one of the parents had education beyond high school) is consistent with several previous studies reporting that people from families with a lower level of education were exposed to more heavy metals [54,63,64,65]. In our previous study, we also found that Jamaican children whose parents both had education levels up to high school had 1.82 times the odds of having a detectable blood arsenic concentration (>1.3 μg/L) than children who had at least one parent with an education level beyond high school (*p* ≤ 0.01) [54]. Jee et al. also demonstrated that a lower level of formal education contributes to significantly higher blood cadmium levels [63]. Another study in Canada reported a significant inverse relationship between education (completed high school or not) and blood mercury levels in pregnant women [65]. A possible reason for our finding is that children from families with a lower level of education tend to be exposed to more fast food that contains a high content of food additives [66]. Moreover, parents with a higher education level may reveal more health-conscious behavior in providing food for their children [67] although they may not be aware that foods such as vegetables, string beans, and lettuce may have high levels of aluminum.

The literature about the association between genetic variation in GST genes and BAlCs in TD children is very limited. Our results support the conclusion that *GSTP1* Ile105Val genotype can modify the effect of consuming saltwater fish on BAlCs where only carriage of the Ile/Ile genotype was shown to confer an increased risk of having a level > 5.0 μg/L. This was observed when either a co-dominant or dominant genetic model was used, while there was no significant association between any of the three GST polymorphisms and BAlCs in the additive models. One mechanism that may help to explain this finding is that codon 105 is located in the active site of the enzyme and that *GSTP1* Ile105Val has been associated with changes in substrate-specific catalytic activity [68,69,70]. Similar relationships have been reported for another heavy metal. Engström et al. found that variation in the amount of fish intake can influence the level of mercury measured in erythrocytes and that this is dependent on *GSTP1* genotype. No significant association with mercury levels was found if fish consumption was low, but individuals with the Ile/Ile genotype had significantly higher mercury levels than those with either the Ile/Val or Val/Val genotype if fish was eaten at least 2.5 times a week [68,71], In addition, previous studies have demonstrated that the GST enzyme itself or glutathione reductase (GR), an enzyme that maintains the supply of the GST substrate reduced glutathione, can be potentially inhibited by heavy metal ions at toxic concentrations [72,73,74]. For example, long term low-level lead exposure in rats has shown significant inhibitory effects (up to 55% inhibition) on GST activity [75]. Cadmium was shown to play a role in the inhibition of GST that was purified from Van Lake fish gills [76]. Since saltwater fish is a source of many heavy metals including arsenic, lead, mercury, and cadmium [77,78], a joint effect of multiple heavy metal exposures through saltwater fish consumption and GST genes is possible in relation to BAlCs. More studies are needed to replicate these relationships.

## 5. Limitations

We acknowledge that this study has several limitations. First, our participants are more likely to be from the Kingston area. Hence, our findings may not be generalizable to all children in Jamaica. Second, the timing of Al exposure was not available in this study as the BAlCs we used as a biomarker are more likely to reflect recent exposure. Although we used a food frequency questionnaire that reflects the food selection in Jamaica, we cannot exclude the possibility that findings may be confounded by other unmeasured variables, such as the consumption of canned beverages or the use of Al foil that may have a strong association with BAlCs. In addition, since we categorized the frequency of food into binary variables (consumed vs. never consumed), our analysis did not account for the quantity of food intake. Furthermore, to avoid the potential multicollinearity, consumption of several food items including freshwater fish, tuna, cakes/buns, vegetables (broad beans, lettuce, cabbage, and root vegetables) that were significantly associated with BAlCs in the univariable analysis were removed from the multivariable analyses. Furthermore, since SES is associated with parental education level, we choose to use parental education level in the model to avoid any potential for multicollinearity. Therefore, we advise caution in interpretation of these findings.

## 6. Conclusions

The present work indicated that children in Jamaica may be more susceptible to Al exposures through specific environmental factors as well as variation in GST genes. Our findings from interactive multivariable logistic regression models revealed that consumption of string beans was associated with higher odds of having a detectable BAlC, whereas higher parental education level was associated with lower odds of having a detectable BAlC in TD children. Additionally, we have found that among children with the *GSTP1* Ile/Ile genotype, the odds of having a detectable BAlC was higher in children who consumed saltwater fish than in those who did not eat such food under both a co-dominant and dominant genetic model for *GSTP1*. This finding suggests that *GSTP1* rs1695 may serve as an effect modifier for the association between saltwater fish consumption and BAlCs in Jamaican children. Further research is recommended to better understand the biological explanation for these findings.

## Figures and Tables

**Table 1:** Characteristics of typically developing (TD) children and their parents (N = 366).

Variables		Categories	n (%)
**Child**	**Sex**	Male	299 (81.7)
		Female	67 (18.3)
	**Age (months)**	Age < 72	275 (75.1)
		Age ≥ 72	91 (24.9)
	**Race**	Afro-Caribbean	356 (97.3)
	**Place** **of birth** **(Parish)**	Kingston parish	229 (62.6)
		Other parishes ^a^	137 (37.4)
**Maternal age (at child’s birth)** **(n = 360)**		Less than 35	319 (88.6)
		More than or equal to 35	41 (11.4)
**Parental education level (n = 356)**		Both up to high school ^b^	194 (54.5)
		At least one beyond high school ^c^	162 (45.5)
**Socioeconomic status (SES)**		High SES (own a car)	149 (40.7)
***GSTT1* (n = 348)**		DD (null alleles)	91 (26.1)
		Homozygote (I/I) or heterozygote (I/D)	257 (73.9)
***GSTM1* (n = 349)**		DD	86 (24.6)
		I/I or I/D	263 (75.4)
***GSTP1* (n = 351)**		Ile/Ile	95 (27.1)
		Ile/Val	179 (51.0)
		Val/Val	77 (21.9)

^a^ Other parishes include all 12 parishes in Jamaica, except for Kingston parish as described previously [58]. ^b^ Up to high school education included Primary/Jr. Secondary, and Secondary/High/Technical schools. ^c^ Beyond high school education included Vocational, Tertiary College, or University.

**Table 2:** Univariable associations of environmental factors and genotypes for GST genes with detectable blood Al concentrations (BAlCs) in typically developing (TD) children (N = 366).

Exposure Variables		Categories		≥LoD (n = 230)	<LoD (n = 136)	Odds Ratio (95% CI)	*p* Value *
**Child**	**Sex**	Male		192 (83.5)	107(78.7)	0.73 (0.43, 1.25)	0.25
	**Age (months)**	Age ≥ 72		62 (27.0)	29 (21.3)	1.36 (0.82, 2.25)	0.23
	**Race**	Afro-Caribbean		223 (97.0)	133 (97.8)	0.72 (0.18, 2.83)	0.64
	**Place of birth** **(Parish)**	Kingston parish		144 (62.6)	85 (62.5)	1.01 (0.65, 1.56)	0.98
**Maternal age in years** **(at child’s birth)**		More than or equal to 35		26 (11.6) ^a^	15 (11.1) ^b^	1.05 (0.53, 2.05)	0.90
**Parental education level**		At least one beyond high school **		89 (39.6) ^c^	73 (55.7) ^d^	0.52 (0.34, 0.80)	0.003
**Socioeconomic status (SES)**		High SES (own a car)		92 (40.0)	57 (41.9)	0.92 (0.60, 1.42)	0.72
*GSTT1* *** ≥LoD (n = 218) <LoD (n = 130)		DD		50 (22.9)	41 (31.5)	0.65 (0.10, 1.05)	0.08
		I/I or I/D		168 (77.1)	89 (68.5)	(ref)	
*GSTM1* *** ≥LoD (n = 218) <LoD (n = 131)		DD		50 (22.9)	36 (27.5)	0.78 (0.48, 1.29)	0.34
		I/I or I/D		168 (77.1)	95(72.5)	(ref)	
***GSTP1*** ≥LoD (n = 219) <LoD (n = 132)		Ile/Ile		61 (27.8)	34 (25.8)	(ref)	
		Ile/Val		111 (50.7)	68 (51.5)	0.91 (0.54, 1.53)	0.72
		Val/Val		47 (21.5)	30 (22.7)	0.87 (0.47, 1.62)	0.67
**Source of Piped water**		Drinking		199 (94.3) ^e^	150 (97.4)	0.76 (0.26, 2.22)	0.61
		Cooking		201 (95.3) ^f^	152 (98.7)	0.55 (0.15, 2.07)	0.38
**Consumption**	**Seafood**	Saltwater fish		160 (75.5)	91 (59.1)	1.40 (0.89, 2.20)	0.14
		Fresh water fish (Pond fish, tilapia)		75 (35.4)	39 (25.3)	1.80 (1.11, 2.91)	0.02
		Tuna (Canned fish)		84 (39.6)	48 (31.2)	0.64 (0.41, 0.99)	0.04
	**Grain and starches**	Whole wheat bread		142 (67.0)	92 (59.7)	1.49 (0.96, 2.31)	0.07
		Cakes/Buns		186 (87.7)	124 (80.5)	1.87 (1.05, 3.32)	0.03
		Pasta, macaroni, noodles		176 (83.0)	141 (91.6)	0.50 (0.25, 1.01)	0.05
	**Fruits and vegetables**	Peas, beans, nut, legumes	Red peas, gungo peas	182 (85.9)	108 (70.1)	1.96 (1.18, 3.27)	0.01
			Broad beans	151 (71.2)	66 (42.9)	1.84 (1.19, 2.83)	<0.01
			String beans	112 (52.8)	42 (27.3)	3.27 (2.05, 5.22)	<0.01
		Root vegetables	Yam, sweet potato, dasheen, coco	140 (66.0)	113 (73.4)	0.60 (0.37, 0.97)	0.03
		Leafy vegetables	Lettuce	146 (68.9)	81 (52.6)	1.84 (1.19, 2.84)	<0.01
			Callaloo, broccoli, or pakchoi	186 (87.7)	112 (72.7)	1.54 (0.90, 2.62)	0.11
			Cabbage	120 (56.6)	108 (70.1)	0.50 (0.32, 0.80)	<0.01
		Fruits	Tomatoes	172 (81.1)	100 (64.9)	1.36 (0.84, 2.19)	0.21
			Ackee	142 (67.0)	110 (71.4)	0.66 (0.41, 1.06)	0.09
			Avocado	151 (71.2)	71 (46.1)	1.51 (0.98, 2.33)	0.06
			Green banana	141 (66.5)	117 (76.0)	0.59 (0.36, 0.95)	0.03
			Fried plantain	183 (86.3)	128 (83.1)	0.59 (0.31, 1.11)	0.10

* *p* values are based on the Wald’s test in logistic regression models. ** Beyond high school education included Vocational, Tertiary College, or University. *** DD, I/I, and I/D are defined for *GSTT1* and *GSTM1* in Table 1. Number of missing data for child’s BAlC ≥ LoD; ^a^ = 5, ^c^ = 5, ^e^ = 1, ^f^ = 1. Number of missing data for child’s BAlC < LoD; ^b^ = 1, ^d^ = 5. (ref) = reference.

**Table 3:** Associations between GST genes and binary detectable blood Al concentrations (BAlCs) based on logistic regression models that include a two-way gene*gene interaction (N = 366).

Models	*GSTT1* ^a,b^ Genotypes	*GSTM1*^c,d^ Genotypes	*GSTP1*^e,f^ Genotypes	OR (95%CI)	*p* Value *	Overall Interaction *p* Value **
**Unadjusted Model for interactive effect between *GSTT1* and *GSTM1* including the corresponding main effect**						
**Recessive**	DD	DD vs. I/I or I/D (ref)		0.43 (0.17, 1.11)	0.08	0.11
	I/I or I/D			1.08 (0.59, 2.00)	0.79	
	DD vs. I/I or I/D (ref)	DD		0.33 (0.13, 0.87)	0.03	
		I/I or I/D		0.84 (0.47,1.48)	0.79	
**Unadjusted Model for interactive effect between *GSTM1* and *GSTP1* including the corresponding main effect**						
**Co-dominant**		DD	Ile/Val vs. Ile/Ile (ref)	0.38 (0.12, 1.19)	0.10	0.06
			Val/Val vs. Ile/Ile (ref)	0.20 (0.05, 0.82)	0.03	
			Ile/Val vs. Val/Val (ref)	1.93 (0.59, 6.28)	0.28	
		I/I or I/D	Ile/Val vs. Ile/Ile (ref)	1.19 (0.66, 2.15)	0.57	
			Val/Val vs. Ile/Ile (ref)	1.32 (0.65, 2.69)	0.45	
			Ile/Val vs. Val/Val (ref)	0.90 (0.47, 1.72)	0.75	
		DD vs. I/I or I/D (ref)	Ile/Ile	2.24 (0.74, 6.74)	0.15	
			Ile/Val	0.71 (0.36, 1.40)	0.32	
			Val/Val	0.33 (0.10, 1.07)	0.06	
**Dominant**		DD	Val/Val or Ile/Val vs. Ile/Ile (ref)	0.32 (0.11, 0.98)	<0.05	0.04
		I/I or I/D		1.23 (0.70, 2.14)	0.47	
		DD vs. I/I or I/D (ref)	Val/Val or Ile/Val	0.59 (0.33, 1.05)	0.07	
			Ile/Ile	2.24 (0.74, 6.74)	0.15	
**Recessive**		DD	Val/Val vs. Ile/Ile or Ile/Val (ref)	0.39 (0.12, 1.23)	0.11	0.10
		I/I or I/D		1.18 (0.64, 2.17)	0.59	
		DD vs. I/I or I/D (ref)	Val/Val	0.33 (0.10, 1.07)	0.06	
			Ile/Ile or Ile/Val	0.99 (0.57, 1.76)	0.99	

DD, I/I, and I/D are defined for *GSTT1* and *GSTM1* in Table 1. * *p* values are based on the Wald’s test in logistic regression models. ** Overall interaction *p* values are based on the type 3 effect test in logistic regression models. Number of missing data are for child’s BAlC ≥ LoD; ^a^ = 12, ^c^ = 12, ^e^ = 11. Number of missing data for child’s BAlC < LoD; ^b^ = 6, ^d^ = 5, ^f^ = 4. (ref) = reference.

**Table 4:** Multivariable logistic regression analysis of associations between children’s exposure to environmental factors and binary detectable blood Al concentrations (BAlCs) by genotypes for GST genes that include gene*environment interaction (N = 366).

Environmental Factor (Food Consumption) (Yes vs. No)	Gene	Models	Genotypes	OR (95% CI)	*p* Value ^a^	Overall Interaction *p* Value ^b^
**Porridge**	*GSTT1*	Recessive	DD	1.51 (0.46, 4.91)	0.49	0.03
			I/I or I/D	0.27 (0.10, 0.73)	0.01	
**Macaroni**	*GSTT1*	Recessive	DD	1.97 (0.52, 7.52)	0.32	0.03
			I/I or I/D	0.22 (0.05, 0.97)	<0.05	
**Green banana**	*GSTT1*	Recessive	DD	1.47 (0.55, 3.90)	0.44	0.04
			I/I or I/D	0.45 (0.25, 0.82)	0.01	
**Broad beans** **(fava beans)**	*GSTM1*	Recessive	DD	3.96 (1.57, 9.97)	<0.01	<0.05
			I/I or I/D	1.37 (0.82, 2.27)	0.23	
**Saltwater fish**	*GSTP1*	Co-dominant	Ile/Ile	3.36 (1.37, 8.24)	<0.01	0.03
			Ile/Val	1.26 (0.67, 2.38)	0.47	
			Val/Val	0.53 (0.18, 1.58)	0.26	
		Dominant	Val/Val or Ile/Val	1.0 (0.58, 1.72)	0.99	0.02
			Ile/Ile	3.36 (1.37, 8.24)	<0.01	
		Recessive	Val/Val	0.53 (0.18, 1.58)	0.26	0.05
			Ile/Ile or Ile/Val	1.76 (1.05, 2.94)	0.03	
**White bread**	*GSTP1*	Co-dominant	Ile/Ile	2.49(1.05, 5.90)	0.04	0.06
			Ile/Val	0.82 (0.43, 1.55)	0.53	
			Val/Val	0.59 (0.20, 1.75)	0.34	
		Dominant	Val/Val or Ile/Val	0.75 (0.43, 1.29)	0.30	0.02
			Ile/Ile	2.49 (1.05, 5.90)	0.04	
		Recessive	Val/Val	0.59 (0.20, 1.75)	0.34	0.24
			Ile/Ile or Ile/Val	1.20 (0.73, 2.00)	0.47	
**Whole wheat bread**	*GSTP1*	Co-dominant	Ile/Ile	0.59 (0.23, 1.52)	0.27	0.07
			Ile/Val	2.19 (1.17, 4.11)	0.01	
			Val/Val	1.76 (0.70, 4.48)	0.23	
		Dominant	Val/Val or Ile/Val	2.05 (1.22, 3.45)	<0.01	0.02
			Ile/Ile	0.59 (0.23, 1.52)	0.27	
		Recessive	Val/Val	1.76 (0.70, 4.48)	0.23	0.72
			Ile/Ile or Ile/Val	1.45 (0.87, 2.42)	0.16	

Number of missing data are based on numbers reported in Table 3 for *GSTT1*, *GSTM1*, and *GSTP1*. DD, I/I, and I/D are defined for *GSTT1* and *GSTM1* in Table 1. Results ^a^ *p* values and ^b^ Overall interaction *p* values are described in Table 3.

**Table 5:** Adjusted associations between exposure to environmental factors and binary detectable blood Al concentrations (BAlCs) by genotypes for *GSTP1* genes in typically developing children based on logistic regression models that include gene*environment interaction (N = 366).

Models for *GSTP1* *	Environmental Factor (EF)	Category	Genotypes	OR (95%CI)	*p* Value ^a^	Overall Interaction *p* Value ^b^
**Co-dominant**	EF1	G1 vs. G2	-	0.57 (0.36, 0.91)	0.02	-
	EF2	Yes vs. No	-	3.07 (1.85, 5.09)	<0.01	-
	EF3	Yes vs. No	Ile/Ile	2.73 (1.07, 6.96)	0.04	0.02
			Ile/Val	0.98 (0.50, 1.93)	0.95	
			Val/Val	0.36 (0.11, 1.14)	0.08	
**Dominant**	EF1	G1 vs. G2	-	0.56 (0.36, 0.90)	0.02	-
	EF2	Yes vs. No	-	2.94 (1.78, 4.83)	<0.01	-
	EF3	Yes vs. No	Ile/Val or Val/Val	0.75 (0.42, 1.35)	0.34	0.02
			Ile/Ile	2.74 (1.08, 6.99)	0.03	
**Recessive**	EF1	G1 vs. G2	-	0.57 (0.36, 0.91)	0.02	-
	EF2	Yes vs. No	-	3.07 (1.85, 5.06)	<0.01	-
	EF3	Yes vs. No	Val/Val	0.36 (0.11, 1.14)	0.08	0.04
			Ile/Ile or Ile/Val	1.39 (0.81, 2.41)	0.23	

* *GSTP1* missing data are based on numbers reported in Table 3. EF1 = Parental education level (Parental education level missing data are based on numbers reported in Table 1). EF2 = Consumption of string beans. EF3 = Consumption of saltwater fish. G1 = beyond high school education included Vocational, Tertiary College, or University. G2 = Primary/Jr. Secondary, and Secondary/High/Technical schools. ^a^ *p* and ^b^ Overall interaction *p* values are described in Table 3.

## Data Availability

The data analyzed in this study are from two grants (i.e., R21 and R01). The data from R01 are or will be publicly available through the National Database for Autism Research (NDAR). Data from R21 will also be available upon request from the corresponding author.

## Supplementary Materials

The following supporting information can be downloaded at: https://www.mdpi.com/article/10.3390/genes13101907/s1, Table S1: Associations between children’s genotypes for *GST* genes and binary detectable blood Al concentrations (BAlCs) by children’s exposure to environmental factors based on logistic regression models that include interaction between *GST* genes and the main environmental exposure (N = 366); Table S2: Associations between children’s genotypes for *GSTP1* and binary detectable blood Al concentrations (BAlCs) by saltwater fish consumption based on logistic regression models that adjusted for parental education level and consumption of string beans (N = 366).
