# Supplementary material for: Interactions between Environmental Factors and Glutathione S-Transferase (GST) Genes with Respect to Detectable Blood Aluminum Concentrations in Jamaican Children
Source: Genes (Basel). 2022 Oct 20;13(10):1907. doi: 10.3390/genes13101907 (PMC9601654; doi:10.3390/genes13101907)
Supplement: Supplementary file 1 [file genes-13-01907-s001.zip › genes-1866596-supplementary.pdf]

genes

Supplementary Materials

Interactions between Environmental Factors and Glutathione S-Transferase (GST) Genes with Respect to Detectable Blood Aluminum Concentrations in Jamaican Children

Mohammad H. Rahbar [1,2,3,*], Maureen Samms-Vaughan [4], Yuansong Zhao [2,5], Sepideh Saroukhani [2,3], Jan Bressler [1,6], Manouchehr Hessabi [2], Megan L. Grove [1,6], Sydonnie Shakespeare-Pellington [4] and Katherine A. Loveland [7]

[1] Department of Epidemiology, Human Genetics, and Environmental Sciences (EHGES), School of Public Health, The University of Texas Health Science Center at Houston, Houston, TX 77030, USA
[2] Biostatistics/Epidemiology/Research Design (BERD) Component, Center for Clinical and Translational Sciences (CCTS), The University of Texas Health Science Center at Houston, Houston, TX 77030, USA
[3] Division of Clinical and Translational Sciences, Department of Internal Medicine, McGovern Medical School, The University of Texas Health Science Center at Houston, Houston, TX 77030, USA
[4] Department of Child & Adolescent Health, The University of the West Indies (UWI), Mona Campus, Kingston 7, Jamaica
[5] Department of Biostatistics & Data Science, School of Public Health, The University of Texas Health Science Center at Houston, Houston, TX 77030, USA
[6] Human Genetics Center, School of Public Health, The University of Texas Health Science Center at Houston, Houston, TX 77030, USA
[7] Louis A Faillace, MD, Department of Psychiatry and Behavioral Sciences, McGovern Medical School, The University of Texas Health Science Center at Houston, Houston, TX 77054, USA
* Correspondence: mohammad.h.rahbar@uth.tmc.edu; Tel.: +1-713-500-7901; Fax: +1-713-500-0766

Citation: Rahbar, M.H.; Samms-Vaughan, M.; Zhao, Y.; Saroukhani, S.; Bressler, J.; Hessabi, M.; Grove, M.L.; Shakespeare-Pellington, S.; Loveland, K.A. Interactions between Environmental Factors and Glutathione S-Transferase (GST) Genes with Respect to Detectable Blood Aluminum Concentrations in Jamaican Children. *Genes* **2022**, *13*, 1907. https://doi.org/10.3390/genes13101907

Academic Editor: Derek Morris

Received: 29 July 2022
Accepted: 18 October 2022
Published: 20 October 2022

Publisher's Note: MDPI stays neutral with regard to jurisdictional claims in published maps and institutional affiliations.

Copyright: © 2022 by the authors. Licensee MDPI, Basel, Switzerland. This article is an open access article distributed under the terms and conditions of the Creative Commons Attribution (CC BY) license (https://creativecommons.org/licenses/by/4.0/).

Table S1. Associations between children's genotypes for GST genes and binary detectable blood Al concentrations (BAlCs) by children's exposure to environmental factors based on logistic regression models that include interaction between GST genes and the main environmental exposure (N = 366).

Gene	Models	Genotypes Compared	Referent Genotypes	Environmental factor (food consumption)	Category	OR (95%CI)	*p* Value [a]	Overall interaction *p* Value [b]
GSTT1 [c]	Recessive	DD [e]	I/I or I/D [f]	Porridge	Yes	0.75 (0.45, 1.26)	0.28	0.03
					No	0.08 (0.01, 0.58)	0.01	
GSTT1 [c]	Recessive	DD [e]	I/I or I/D [f]	Macaroni	Yes	0.78 (0.47, 1.33)	0.37	0.03
					No	0.14 (0.04, 0.61)	0.01	
GSTT1 [c]	Recessive	DD [e]	I/I or I/D [f]	Green banana	Yes	0.89 (0.51, 1.56)	0.68	0.04
					No	0.27 (0.10, 0.74)	0.01	
GSTM1 [d]	Recessive	DD [e]	I/I or I/D [f]	Broad beans (fava beans)	Yes	1.18 (0.60, 2.31)	0.63	<0.05
					No	0.41 (0.18, 0.92)	0.03	
GSTP1 [g]	Co-dominant	Ile/Val	Ile/Ile	Saltwater fish	Yes	0.64 (0.33, 1.25)	0.19	0.03
					No	1.70 (0.71, 4.07)	0.23	
		Val/Val	Ile/Ile		Yes	0.48 (0.22, 1.04)	0.06	
					No	3.04 (0.93, 9.89)	0.06	
		Ile/Val	Val/Val		Yes	1.33 (0.69, 2.53)	0.39	
					No	0.56 (0.19, 1.64)	0.29	
	Dominant	Val/Val or Ile/Val	Ile/Ile	Saltwater fish	Yes	0.58 (0.31, 1.10)	0.1	0.02
					No	1.96 (0.85, 4.52)	0.12	
	Recessive	Val/Val	Ile/Ile or Ile/Val	Saltwater fish	Yes	0.65 (0.35, 1.20)	0.17	0.05
					No	2.14 (0.76, 6.02)	0.15	
	Co-dominant	Ile/Val	Ile/Ile	White bread	Yes	0.58 (0.29, 1.15)	0.12	0.06
					No	1.77 (0.77, 4.04)	0.18	
		Val/Val	Ile/Ile		Yes	0.52 (0.24, 1.14)	0.10	

				No	2.21 (0.70, 7.00)	0.18	
	Ile/Val	Val/Val		Yes	1.11 (0.58, 2.11)	0.76	
				No	0.80 (0.27, 2.37)	0.69	
Dominant	Val/Val or Ile/Val	Ile/Ile	White bread	Yes	0.56 (0.29, 1.08)	0.08	0.02
				No	1.86 (0.84, 4.10)	0.12	
Recessive	Val/Val	Ile/Ile or Ile/Val	White bread	Yes	0.76 (0.41. 1.40)	0.38	0.24
				No	1.55 (0.55, 4.39)	0.40	
Co-dominant	Ile/Val	Ile/Ile	Whole wheat bread	Yes	1.43 (0.76, 2.68)	0.27	0.07
				No	0.38 (0.15, 0.99)	0.047	
	Val/Val	Ile/Ile		Yes	1.30 (0.59, 2.87)	0.52	
				No	0.43 (0.15, 1.26)	0.12	
	Ile/Val	Val/Val		Yes	1.10 (0.53, 2.29)	0.8	
				No	0.88 (0.38, 2.06)	0.77	
Dominant	Val/Val or Ile/Val	Ile/Ile	Whole wheat bread	Yes	1.39 (0.77, 2.52)	0.28	0.02
				No	0.40 (0.16, 0.98)	<0.05	
Recessive	Val/Val	Ile/Ile or Ile/Val	Whole wheat bread	Yes	1.04 (0.52, 2.08)	0.91	0.72
				No	0.86 (0.38, 1.92)	0.70	

[a] *p* values are based on the Wald's test in logistic regression models. [b] Overall interaction *p* values based on the type 3 effect test in logistic regression models. [c] *GSTT1* was missing for 12 children with BAlCs above LoD and 6 children with BAlCs below LoD. [d] *GSTM1* was missing for 12 children with BAlCs above LoD and for 5 children with BAlCs below LoD. [e] DD indicates the null alleles for *GSTT1*and *GSTM1*. [f] I/I or I/D indicate the homozygote (I/I) or a heterozygote (I/D) for *GSTT1* and *GSTM1*. [g] *GSTP1* was missing for 11 children with BAlCs above LoD and 4 children with BAlCs below LoD.

Table S2. Associations between children's genotypes for *GSTP1* and binary detectable blood Al concentrations (BAlCs) by saltwater fish consumption based on logistic regression models that adjusted for parental education level and consumption of string beans (N = 366).

Models	Environmental factor (food consumption)	Category	Gene	Genotypes	OR (95%CI)	*p* Value [a]	Overall interaction *p* Value [b]
**Co-dominant**	Saltwater fish	Yes	*GSTP1*[c]	Ile/Val vs. Ile/Ile (ref)	0.59 (0.29, 1.21)	0.15	0.02
				Val/Val vs. Ile/Ile (ref)	0.41 (0.18, 0.94)	0.03	
				Ile/Val vs. Val/Val (ref)	1.46 (0.72, 2.95)	0.30	
		No		Ile/Val vs. Ile/Ile (ref)	1.66 (0.67, 4.10)	0.28	
				Val/Val vs. Ile/Ile (ref)	3.12 (0.92, 10.58)	0.07	
				Ile/Val vs. Val/Val (ref)	0.53 (0.17, 1.63)	0.27	
**Dominant**	Saltwater fish	Yes	*GSTP1*[c]	Val/Val or Ile/Val vs. Ile/Ile (ref)	0.53 (0.27, 1.04)	0.06	0.02
		No			1.94 (0.82, 4.60)	0.13	
**Recessive**	Saltwater fish	Yes	*GSTP1*[c]	Val/Val vs. Ile/Ile or Ile/Val (ref)	0.58 (0.30, 1.13)	0.11	0.04
		No			2.26 (0.77, 6.59)	0.14	

[a] *p* values are based on the Wald's test in logistic regression models. [b] Overall interaction *p* values based on the type 3 effect test in logistic regression models. [c] *GSTP1* was missing for 11 children with BAlCs above LoD and 4 children with BAlCs below LoD.
